# Evaluating the Effect of Non-cellular Bioactive Glass-Containing Scaffolds on Osteogenesis and Angiogenesis in *in vivo* Animal Bone Defect Models

**DOI:** 10.3389/fbioe.2020.00430

**Published:** 2020-05-14

**Authors:** Chanuka D. S. Ranmuthu, Charindu K. I. Ranmuthu, Jodie C. Russell, Disha Singhania, Wasim S. Khan

**Affiliations:** ^1^Cambridge Clinical School, University of Cambridge, Cambridge, United Kingdom; ^2^Division of Trauma and Orthopaedics, Department of Surgery, Addenbrooke's Hospital, University of Cambridge, Cambridge, United Kingdom

**Keywords:** scaffolds, bone, bioactive glass, osteogenesis, angiogenesis

## Abstract

The use of bone scaffolds to replace injured or diseased bone has many advantages over the currently used autologous and allogeneic options in clinical practice. This systematic review evaluates the current evidence for non-cellular scaffolds containing bioactive glass on osteogenesis and angiogenesis in animal bone defect models. Studies that reported results of osteogenesis via micro-CT and results of angiogenesis via Microfil perfusion or immunohistochemistry were included in the review. A literature search of PubMed, EMBASE and Scopus was carried out in November 2019 from which nine studies met the inclusion and exclusion criteria. Despite the significant heterogeneity in the composition of the scaffolds used in each study, it could be concluded that scaffolds containing bioactive glass improve bone regeneration in these models, both by osteogenic and angiogenic measures. Incorporation of additional elements into the glass network, using additives, and using biochemical factors generally had a beneficial effect. Comparing the different compositions of non-cellular bioactive glass containing scaffolds is however difficult due to the heterogeneity in bioactive glass compositions, fabrication methods and biochemical additives used.

## Introduction

Bones are composed of a dense connective tissue and they serve a large variety of functions. These include mechanical functions such as internal organ protection, synthetic functions such as hematopoiesis within the bone marrow and metabolic functions such as acting as reservoirs for minerals (Taichman, 2005). Thus, the repair of bone defects caused by trauma, infection and congenital abnormalities is important (Zhao et al., 2015). Many methods to achieve regeneration have been investigated, including the “gold standard” autologous bone grafting, allograft implantation, or autologous bone transplantation. However, these methods are not without their limitations, especially with regards to cost-effectiveness and even efficacy (Dimitriou et al., 2011). One of the alternative methods proposed is to focus on regenerating the tissue through tissue engineering, rather than replacing it (Langer, 2000). Commonly in tissue engineering, templates or “scaffolds” are used to provide an appropriate environment for this tissue regeneration (O'Brien, 2011). Scaffolds can be seeded with cells and growth factors in order to aid this process.

The ideal properties of a scaffold have been described previously (O'Brien, 2011). Firstly, the scaffold must be biocompatible, allowing for cells to migrate through the scaffold and lay down new matrix. They must elicit a negligible immune reaction to prevent any inflammatory process from reducing healing of the bone. They should also be biodegradable, with degradation products being non-toxic. Having both mechanical properties and sufficient porosity to function adequately from the implantation to remodeling stage is also important. An interconnected pore structure within the scaffold is crucial to facilitate the penetration of cells and diffusion of nutrients. Research suggests that the minimum pore size for bone regeneration is 100 μm (Wagoner Johnson and Herschler, 2011).

Other properties described (Wagoner Johnson and Herschler, 2011) include the ability to serve as a template for bone formation by allowing cells to adhere and proliferate (osteoconductivity). They should also be able to bind with surrounding bone tissue (bioactivity) and ideally have the ability to induce bone formation (osteoinductivity). Furthermore, a strong correlation between decreased vascular function and failed bone healing has been described in the literature previously (Dickson et al., 1994). The importance of re-establishing an adequate blood vessel system has been comprehensively reviewed previously (Stegen et al., 2015). Indeed, the low survival of osteogenic cells after implantation of the scaffold in the early stages of bone regeneration has been attributed to an inadequate rate of blood vessel invasion into the scaffold (Giannoni et al., 2010; Stegen et al., 2015). Thus, scaffolds that have potential to provide better angiogenesis are also sought after (Rouwkema and Khademhosseini, 2016).

A plethora of scaffolds made from different biomaterial groups have been investigated in the field, including but not limited to calcium phosphate bioactive ceramic scaffolds, polymeric scaffolds, composite scaffolds, and metallic scaffolds (Bose et al., 2012). Bioactive glass scaffolds have also recently gained attention. The first bioactive glass introduced was the 45S5 Bioglass® introduced by Hench et al. (1971) and was composed of a quaternary SiO_2_-CaO–Na_2_O–P_2_O_5_ oxide system. It was “bioactive” in the sense that it could form a hydroxycarbonated apatite (HCA) layer on the glass surface upon contacting solutions mimicking human plasma, allowing for a bonding interface with the bone tissue (Hench, 1991). Many other bioactive glasses have similar oxide compositions to the original 45S5 Bioglass®, but with varying concentrations (Brauer, 2015)—some such compositions reviewed in this article are displayed in Table 1. One of the key features of bioactive glasses are that they release ionic dissolution products which can control osteoblastic gene expression, allowing for their bone regenerative ability (Xynos et al., 2001). Further, bioactive glass is able to release soluble ions (such as Si, Ca, P, Na ions) at the rate required for proliferation and differentiation of cells (Larry and Hench, 2006; El-Rashidy et al., 2017). Previous investigations have established that they are osteoconductive and osteoinductive too (Hench et al., 2014). Recently, bioactive glass has gained traction because of its ability to induce the angiogenesis needed for bone regeneration, as aforementioned (Kargozar et al., 2018a). This could provide a more cost-effective way of promoting neovascularization than the use of growth factors (Rahaman et al., 2011). For example, some bioactive glasses such as S53P4 (53% SiO_2_, 23% Na_2_O, 20% CaO, 4% P_2_O_5_) have been shown to increase vascular endothelial growth factor (VEGF) secretion to stimulate angiogenesis in bone (Detsch et al., 2014). Authors have highlighted that investigating ways of the promoting of angiogenesis as one of the most important topics in regenerative medicine (Rouwkema and Khademhosseini, 2016). Thus bioactive glasses may provide one such solution (Kargozar et al., 2018a).

**Table 1:** Study characteristics.

**Paper**	**Glass composition**	**Glass fabrication method**	**Scaffold fabrication method**	**Animal**	**Animal bone defect**	**Added components to scaffold**	**Number of defects**	**Groups**
Jing et al. (2018)	Bioactive glass: 45% SiO_2_, 24.5% Na_2_O, 24.5% CaO and 6% P_2_O_5_ by percentage weight	Not specified. The bioactive glass was sourced from a commercial source (Hubei Central China Medical Materials Co Ltd.)	45S5 Bioglass-based scaffolds fabricated by the foam replication method. The porous scaffolds were loaded with Icariin, sterilized with ultraviolet light and dried in a sterile environment before cell seeding	Rat	Skull	None	1 circular calvarial defect with a diameter of 8mm in 20 rats	1. Negative Control 2. 45S5 bioactive glass 3. 45S5 bioactive glass/autologous stem cells (not relevant for this study) 4. 45S5 bioactive glass/autologous stem cells (not included in this study) 5. Icariin/45S5 bioactive glass scaffold/autologous stem cells (not relevant for this study)
Wang et al. (2019)	MBG: 80% Si, 15% Ca, 5% P by percentage mol	MBG: P123 (4.0 g), TEOS (6.7 g), Ca(NO_3_)2∙4H_2_O (1.4 g), TEP (0.36 g) with molar ratio of Si:Ca:P = 80:15:5	MBG-GO scaffolds were calcined at 500°C under nitrogen protection for 5 h The scaffolds were sterilized using gamma irradiation	Rat	Skull	None	2 critical-sized calvarial defects with a diameter of 5 mm in 24 rats	1. MBG scaffold 2. MBG-LGO scaffold (low graphene oxide) 3. MBG-HGO scaffold (high graphene oxide)
Wu et al. (2019)	Bioactive glass: 95% SiO_2_, 2.5% CaO, 2.5% CuO by percentage mol	Cu-BG NPs with designed compositions and sizes were synthesized via a modified Stöber method	Cu-BG NPs were incorporated into chitosan (CH)/silk fibroin (SF)/glycerophosphate (GP) composites	Rat	Skull	Chitosan/silk fibroin composite	2 full-thickness calvarial bone defects with diameters of 5 mm in 30 rats	1. Chitosan-silk fibroin- glycerophosphate 2. Bioactive glass- Chitosan-silk fibroin- glycerophosphate 3. Copper/Bioactive glass-Chitosan+ silk fibroin-glycerophosphate (1st concentration) 4. Copper/Bioactive glass-Chitosan+ silk fibroin-glycerophosphate (2nd concentration)
Min et al. (2015)	MBG: 80% SiO_2_, 15% CaO, 5% P_2_O_5_ by percentage mol	MBG synthesized by using non-ionic block copolymers as structure-directing agents through an EISA process The dried gel was calcined at 700 °C for 5 h to obtain the final MBG products	DMOG delivering scaffold composed of MBG and PHBHHx polymers were fabricated using a 4th generation 3D-Bioplotter system	Rat	Skull	DMOG and MBG with PHBHHx polymers (MPHS scaffolds)	2 critical-sized bone defects with a diameter of 5 mm in 12 rats	1. MPHS 2. MPHS/DMOG
Xin et al. (2017)	MBG: 80% SiO_2_, 16% CaO, 4% P_2_O_5_ by percentage mol	MBG synthesized by a modified Stöber method. MBG nanoparticles were obtained after removing the templates and organic components by sintering in air at 650°C for 3 h (2°C per min)	MBGNs chemically modified with photo-cross-linkable GelMA were further incorporated into GelMA to fabricate GelMA-G-MBGNs	Rat	Skull	Photo-cross-linkable GelMA + GelMA	1 critical-sized bone defect with a diameter of 5 mm in 6 rats	1. Negative Control without scaffold 2. GelMA (not relevant for this study) 3. GelMA/MBGNs 4. GelMA-G-MBGNs
Qi et al. (2017)	MBG: 80% Si, 15% Ca, 5% P by percentage mol	MBG synthesized by using non-ionic block copolymers as structure-directing agents through EISA process. The dried gel was calcined at 700 °C for 5 h to obtain the final MBG products	MBG-PHBHHx composite scaffolds were prepared by freeze-drying and a particulate leaching technique	Rat	Skull	DMOG + rhBMP-2	2 critical-sized bone defects with a diameter of 5mm in 24 rats	1. Pure MBG-PHBHHx = PHMG 2. BMP-2/MBG-PHBHHx = PHMB 3. DMOG/MBG-PHBHHx = PHMD 4. BMP-2/DMOG/MBG-PHBHHx = PHMBD.
Li et al. (2019)	MBG: 80% SiO_2_, 15% CaO, 5% P_2_O_5_	MBG synthesized by using non-ionic block copolymers as structure-directing agents through EISA process for 72 h. The dried gel was then calcined at 700°C for 5 h and thoroughly ground and sieved to obtain MBG powders	Scaffolds consisting of pure PLGA matrix or MBG-incorporated PLGA matrix were fabricated by a supercritical CO_2_ foaming technique	Rat	Skull	Bioactive lipid FTY720	2 critical-sized bone defects with a diameter of 5 mm in 24 rats	1. Negative control 2. PLGA (not relevant for this study) 3. MBG-PLGA 4. FTY/MBG-PLGA
Jia et al. (2015)	1. Silicate 13–93: 54.6% SiO_2_, 6.0% Na_2_O, 7.95% K_2_O, 7.7% MgO, 22.1% CaO, 1.7% P_2_O_5_ by percentage mol. 2. Borosilicate 2B6Sr: 18.0% SiO_2_, 36.0% B_2_O_3_, 6.0% Na_2_O, 8.0% K_2_O, 2.1% MgO, 6.0% SrO, 22.0% CaO, 2.0% P_2_O_5_ by percentage mol	Not specified. The bioactive glass was sourced from a commercial source (SEM-COM Co. Toledo, OH)	Direct ink writing technique was used with glass inks prepared and a robotic deposition device used to extrude the inks through a 250 μm nozzle. After extrusion, the scaffolds were dried in air and then heated at 1°C per min to 600°C to decompose the organic polymers before sintering at 700°C for 1 h (13–93 glass) and 620°C for 2 h (2B6Sr glass)	Rabbit	Femur	None	1 critical-sized defect 10 mm in length in 44 rabbits	1. Negative control without scaffold 2. Autologous bone graft (not relevant for this study) 3. 13–93 glass scaffolds 4. 2B6Sr glass scaffolds
Zhao et al. (2015)	MBG: 57.2% SiO_2_, 7.5% P_2_O_5_, 35.3% (SrO + CaO) by percentage weight	The MBG powders were calcined from room temperature to 650°C with a heating rate of 1°C per min in air, and maintained at 650°C for 6 h to remove the organic structure-directing agents completely	Sr-MBG scaffolds were fabricated using a commercial 3D Bioplotter printing device (EnvisionTEC GmbH, Germany). Cylinder models were loaded and scaffolds printed layer-by-layer through the extrusion of the paste as a fiber	Rat	Skull	Sr	2 critical-sized defects with a diameter of 5mm in 18 rats	1. Negative control without scaffold 2. MBG 3. Sr-MBG

*Summary table detailing author names, publication year, composition of bioactive glass scaffold, glass fabrication method, animals used, bone defects used, added components to the bioactive glass scaffolds, number of bone defects in each animal, and the experimental groups used in each study*.

*45S5, glass with 45 wt.% of SiO_2_ and 5:1 molar ratio of Calcium to Phosphorus; Cu-BG NPs, copper-containing bioactive glass nanoparticles; DMOG, dimethyloxallyl glycine; EISA, Evaporation-Induced Self-Assembly; FTY720, fingolimod; GelMA-G-MBGNs, MBGNs with photo-cross-linkable GelMA which have been further integrated into GelMA; GelMA, gelatin derivative containing gelatin + methacrylicanhydride; PHBHHx, poly(3-hydroxybutyrate-co-3-hydroxyhexanoate); MBG, mesoporous bioactive glasses; GO, graphine oxide; MBGN, mesoporous bioactive glass nanoparticle; MPHS, mesoporous bioactive glass with poly(3-hydroxybutyrate-co-3-hydroxyhexanoate); mol, moles; Mm, millimeters; PLGA, poly (lactic-co-glycolic acid); rhBMP-2, recombinant human bone morphogenetic protein-2; Sr, strontium*.

There has been a recent trend in adding other components to the bioactive glass in order to aid regeneration. It has been suggested that adding suitable dopants could influence osteoblastic differentiation (Nohra et al., 2014). These include metallic elements such as copper, strontium and borosilicate, biochemical factors such as dimethyloxallyl glycine (DMOG) and organic polymers such as photo-cross linkable gelatin derivative containing gelatin and methacrylicanhydride (GeIMA). Khan et al. found that adding strontium promoted early stage *in vivo* osteointegration and bone remodeling (Khan et al., 2016). A previous study, for example, showed that DMOG, an inhibitor of the enzyme hypoxia-inducible factor prolyl hydroxylase (HIF-PH), could mimic a hypoxic microenvironment and thereby increase angiogenic capacity and osteogenic differentiation of human bone marrow stromal cells (Wu et al., 2013a). Other studies have indicated that bioactive glass and GeIMA composites could encourage cell attachment and proliferation as well as osteogenic differentiation.

There is a large body of evidence supporting the efficacy of various types of bioactive glass scaffolds to induce angiogenesis and osteogenesis *in vitro*. For example, Zhang et al. recently found that their bioactive glass functionalized chondroitin sulfate hydrogel had promoted the formation of a vascular network (Zhang et al., 2019). Jia et al. looked at bioactive silicate (13–93) and borosilicate (2B6Sr) glass scaffolds and found that they were able to facilitate cell attachment and promoted formation of VEGF (Jia et al., 2019). They could also help stimulate mineral deposition and osteoblast marker gene expression. Quinlan et al. found that using cobalt bioactive glass with a particular particle size could encourage the formation of VEGF and could also help osteoblast cell proliferation irrespective of particle size *in vitro* (Quinlan et al., 2015). These *in vitro* analyses can provide an evaluation of cell differentiation and proliferation, as well as cytotoxicity (Hanks et al., 1996), and avoid the unnecessary use of animals. However, it is well-documented that *in vitro* studies can only conduct evaluations of toxicity in the short term, due to the short lifespan of cultured cells (Pizzoferrato et al., 1994). Further, *in vitro* evaluation test more simplified parts of the more complex *in vivo* mechanisms due to the fact that *in vivo* cells often interact with another (Pizzoferrato et al., 1994). It is therefore important to evaluate scaffolds *in vivo*.

It is also important that we have viable methods to assess the efficacy of these scaffolds *in vivo* to promote angiogenesis and osteogenesis. Advances in micro-CT has allowed studies to assess the mineralization of bone formation spatially and temporally in a non-destructive way (van Lenthe et al., 2007) and can provide information such as bone mineral density (BMD), bone volume fraction and new bone volume. Immunohistochemistry has allowed studies to investigate angiogenesis. A plethora of growth factors have been shown to promote angiogenesis. For example, VEGF has been shown to stimulate endothelial proliferation and migration (Ferrara et al., 1992). Other endothelial specific markers include CD31 and CD34 (Miettinen et al., 1994). Detection of such markers allow for an easy way to identify blood vessels and endothelial cells (Lu et al., 2006). Another way in which this can be assessed is the use of Microfil® perfusion and subsequent micro-CT. This method enables not only the visualization of blood vessels but the quantitative characterization of blood vessels and their branching (Ehling et al., 2014) and so is considered to be superior to angiography or blood vessel casting (Lu et al., 2006).

Due to the aforementioned advantages of these methods, only studies which included an immunohistochemistry or Microfil approach to assess angiogenesis combined with micro-CT analyses for osteogenesis were included in this study. This provides a more robust assessment of scaffolds and their outcomes of angiogenesis and osteogenesis in these *in vivo* studies. As previously mentioned, several studies in the literature utilize scaffolds which have added biochemical components. Due to the heterogeneity in the components added, few systematic reviews have included such studies. We provide a comprehensive, update on *in vivo* studies looking at bioactive glass- containing non-cellular scaffolds with or without added biochemical components for osteogenesis and angiogenesis.

## Methods

A literature search was performed across three databases, PubMed, Scopus and EMBASE, in November 2019 using the search terms “bioactive glass AND bone AND angiogenesis” from 2015 to present. The inclusion criteria were as follows: (1) *in vivo* animal studies, using surgically-created bone defects, without any pre-treatment, to assess bone regeneration, (2) published in a peer-reviewed journal, (3) published from 2015 onwards, (4) containing study groups with no additional cells seeded in a bioactive glass-containing scaffold, (5) with the scaffold being implanted directly into the bone defect, (6) assessing both angiogenesis and osteogenesis to establish extent of bone regeneration in model, (7) assessing angiogenesis using either immunohistochemistry or Microfil® perfusion method, and (8) assessing osteogenesis using micro-CT method. Exclusion criteria were: (1) all *in vitro* studies, (2) conference papers, (3) studies not published in English, and (4) articles for which the full-text version was not freely accessible.

Title and abstract screening was performed by four authors (CKIR, CDSR, JCR, and DS) on the non-duplicate articles retrieved by the initial search against the inclusion criteria. An additional hand search was also performed on pertinent review articles for any articles that might have been missed by the initial search. Full-text articles were retrieved for those chosen for further assessment.

## Results

**Figure 1:**
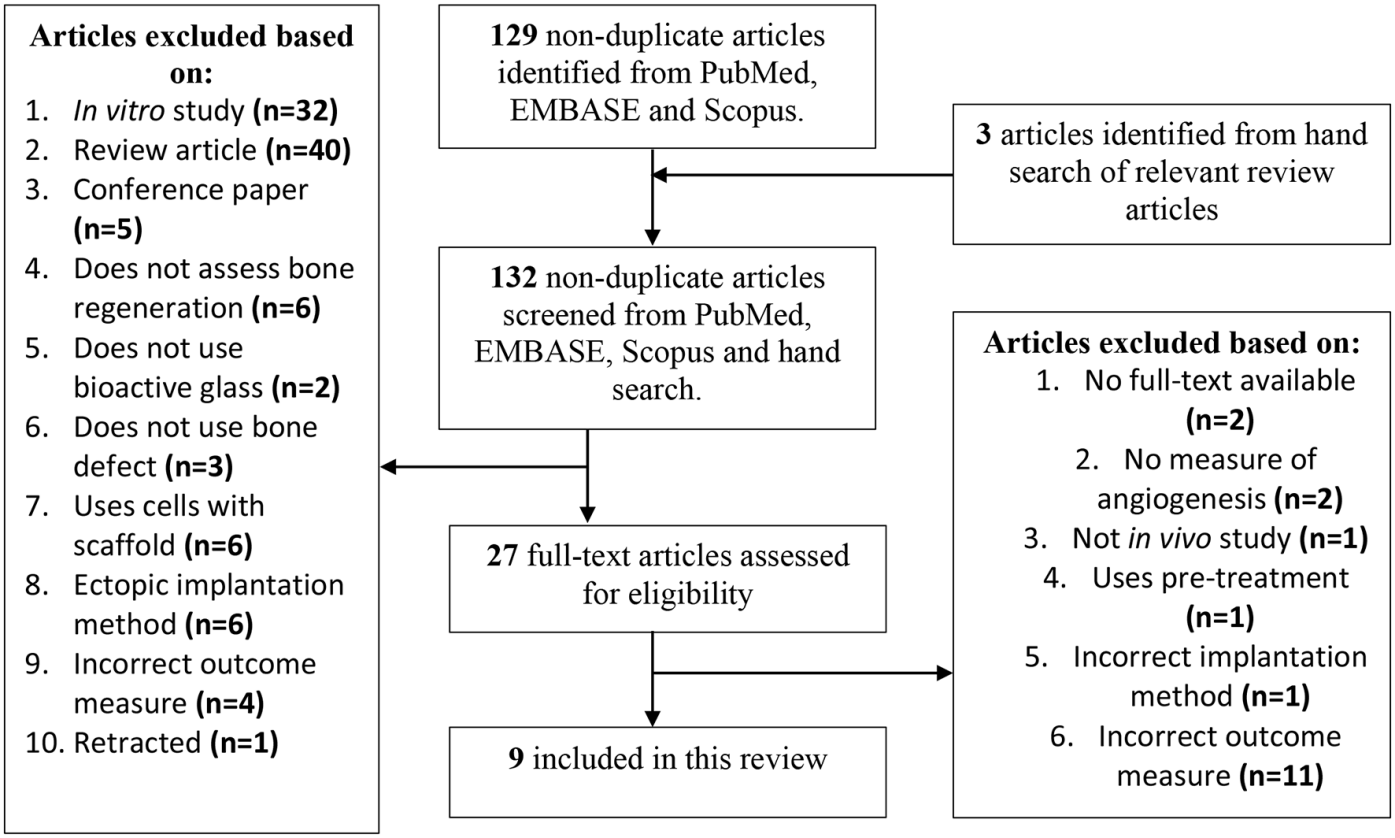
Systematic review study selection flow chart.

The outline of the search results is displayed in Figure 1. To be included in this review, studies must have used bioactive glass-containing scaffolds and implanted them directly into a bone defect. Those that did not directly insert them into a bone defect are referred to as “ectopic implantation method.” Those that treated the bone to cause osteonecrosis before creating the bone defect were also excluded and are referred to as “uses pre-treatment.” Articles must have reported outcomes for both angiogenesis, in the form of either Microfil® perfusion or immunohistochemistry, and osteogenesis, in the form of micro-CT. Those that did not report a measure of angiogenesis are given as “no measure of angiogenesis.” Articles that did not report using the appropriate outcome measures detailed above are given as “incorrect outcome measure.”

The initial search and hand search for this systematic review returned nine studies. The study characteristics of these nine papers are detailed in Table 1. Table 2 shows the results of the studies included in this review.

**Table 2:** Study results.

**Paper**	**Evidence of osteogenesis (micro-CT)**	**Evidence of angiogenesis**	
		**Immunohistochemistry results**	**Microfil^®^ perfusion results**
Jing et al. (2018)	**12W:** in the group using 45S5 Bioglass scaffold alone, new bone was created and partially repaired the bone defect. This was compared to the negative control in which minimal bone was created	**12W: CD31 and VEGF staining:** number of both CD31 and VEGF-positive microvessels were significantly higher with 45S5 bioactive glass scaffold treated defects compared to the negative control experiments (*p* < 0.05)	
Wang et al. (2019)	**12W:** New bone formation was seen in all groups however this was markedly increased in groups treated with bioactive glass scaffolds treated more graphene oxide: **Bone mineral density:** MBG-HGO showed significantly greater BMD (0.64 ± 0.08 g/cm^3^) than MBG group (0.10 ± 0.04 g/cm^3^) and MBG-LGO group (0.50 ± 0.04 g/cm^3^) (*p* < 0.05). MBG-LGO showed significantly greater bone mineral density than the MBG group (*p* < 0.05) **Bone volume fraction:** MBG-HGO showed significantly greater bone volume/total volume than MBG group and MBG-LGO group (*p* < 0.05). MBG-LGO showed significantly greater bone volume/total volume than MBG group (*p* < 0.05)	**4W: CD34 staining:** showed very little staining with pure bioactive glass scaffolds, however, staining was very strong in bioactive glass scaffolds treated with graphene oxide	**12W**: **Microfil**^®^ **perfusion experiments:** showed that there were higher levels of new vascularization with graphene oxide treated bioactive glass scaffolds
Wu et al. (2019)	**8W: Bone mineral density and bone volume fraction:** In comparison to gels without bioactive glass, gels with bioactive glass showed significantly increased levels of osteogenesis as measured by BMD and bone volume fraction. Adding copper to the bioactive glass-silk fibroin-chitosan composite further increased levels of osteogenesis, showing full repair of the defect Cu-BG/CH/SF/GP(II) gel had highest BMD and BV/TV	**8W:** **α** **smooth muscle (α-SMA) antigen staining:** slight staining present in both bioactive glass containing group (BG/CH/SF/GP) and non-bioactive glass containing group (CH/SF/GP). Copper treated bioactive glass containing groups Cu-BG/CH/SF/GP (I) and Cu-BG/CH/SF/GP (II) gel showed significantly more staining than those without copper (BG/CH/SF/GP and CH/SF/GP)	
Min et al. (2015)	**8W:** markedly increased bone growth was observed in defects treated with bioactive glass scaffolds (+/–DMOG loading) compared to controls treated without an implant		**8W: Microfil**^®^ **perfusion experiments:** showed new vascularization in the bone defects implanted with bioactive glass scaffolds (+/–DMOG loading). DMOG loaded bioactive glass scaffolds showed more ingrowth of dense vessels into the of the defect compared to DMOG-unloaded bioactive glass scaffolds, which promoted growth around the periphery
Xin et al. (2017)	**4W and 8W**: volume of new bone and volume of mature bone in bioactive glass containing scaffold groups (GelMA/MBGNs and GelMA-G-MBGNs) was significantly more than in non-bioactive glass containing scaffold groups (GelMA and control groups) **Bone volume fraction:** GelMA-G-MBGNs > GelMA/MBGNs > GelMA.	**4W: CD31 staining:** GelMA-G-MBGNs group > GelMA/MBGNs group > GelMA group > control group when measured at same time interval after implantation (*p* < 0.05)	
Qi et al. (2017)	**8W:** PHMBD > PHMB> PHMD > PHMG and PHMD groups in order of osteogenesis **Bone mineral density:** BMD was greatest for the PHMBD group (0.876 ± 0.021g/cm^3^), which was significantly greater than the PHMG, PHMB and PHMD groups. PHMB group had significantly greater BMD than both PHMG and PHMD groups	**CD31 staining**: greater in PHMD and PHMBD than PHMB and PHMG groups	**8W: Microfil**^®^ **perfusion experiments: New blood vessel areas:** PHMBD (86.09 ± 3.989%) > PHMD (36.11 ± 3.687%) >PHMB (21.648 ± 2.459%) >PHMG groups (1.265 ± 0.415%) (all *p* < 0.05) PHMBD group had the greatest area of neovascularization of defects forming microvessels and good connectivity between vessels. Neovascularization was observed in PMBD and PHMD groups but less in PHMB group and the least in PHMG
Li et al. (2019)	**8W: New bone volume:** FTY/MBG-PLGA (9.15 ± 1.2%, *p* < 0.05) > MBG-PLGA (9.15 ± 1.2%, *p* < 0.05) > PLGA group (all *p* < 0.05). No difference between PLGA and controls **Bone volume fraction:** FTY/MBG-PLGA > MBG-PLGA (*p* < 0.05) FTY/MBG-PLGA > PLGA (*p* < 0.01) FTY/MBG-PLGA> neg control (*p* < 0.001) MBG-PLGA> PLGA (*p* < 0.05) MBG-PLGA> neg control (*p* < 0.01)	**CD31 staining:** FTY/MBG-PLGA > MBG-PLGA (*p* < 0.05) FTY/MBG-PLGA > PLGA (*p* < 0.01) FTY/MBG-PLGA> neg control (*p* < 0.01) MBG-PLGA> PLGA (*p* < 0.05) MBG-PLGA> neg control (*p* < 0.05)	**8W: Microfil**^®^ **perfusion experiments: New blood vessel areas:** FTY/MBG-PLGA (21.07 ± 2.02%) > MBG-PLGA group (10.25 ± 1.26%) > PLGA (4.10 ± 0.84%) -all *p* < 0.05 FTY/MBG-PLGA had greatest area of neovascularization
Jia et al. (2015)	**3M and 9M: new bone formation:** Both silicate 13–93 and borosilicate 2B6Sr showed significantly more new bone formation compared to the negative control **9M:** complete bone healing in both silicate 13–93 and borosilicate 2B6Sr groups compared to negative control where a gap in defect was observed	**CD31 staining:** **3M:** 2B6Sr glass scaffold >13–93 glass scaffold and ABG group (*P* < 0.05) **9M:** no difference between 2B6Sr glass scaffold, 13–93 glass scaffold and ABG groups Increased blood vessels observed from 3M to 9M in 2B6Sr glass scaffold >13–93 glass scaffold and ABG group	
Zhao et al. (2015)	**8W: bone mineral density:** Sr-MBG scaffolds group (503.30 ± 88.93 mg cm^−3^) > MBG group (339.30 ± 36.61 mg cm^−3^) > negative control group (58.67 ± 20.65 mg cm^−3^) (all *p* < 0.05) **Bone volume fraction:** Sr-MBG scaffolds group (31.33 ± 4.93%) > MBG scaffolds group (17.67 ± 5.03%) > negative control group (4.33 ± 1.52%) (all *p* < 0.05)		**8W: Microfil**^®^ **perfusion experiments: New blood vessel areas and vessel number:** Sr-MBG scaffold > MBG scaffold > negative control (all *p* < 0.05)

*Summary table detailing author names, publication date, evidence of osteogenesis measured using micro-CT and evidence of angiogenesis, measured using Microfil® perfusion and immunohistochemistry, after implantation of bioactive glass scaffolds into animal models*.

*45S5, glass with 45 wt.% of SiO_2_ and 5:1 molar ratio of Calcium to Phosphorus; ABG, autologous bone graft; BMP-2, human bone morphogenetic protein-2; BV/TV, bone volume/total bone volume; CD31, cluster of differentiation 31; CD34, cluster of differentiation 34; Cu-BG/CH/SF/GP(II), copper-doped bioactive glass with chitosan (CH)/silk fibroin (SF)/glycerophosphate (GP) concentration 2; Cu-BG/CH/SF/GP, copper-doped bioactive glass with chitosan (CH)/silk fibroin (SF)/glycerophosphate (GP) concentration 1; DMOG, dimethyloxallyl glycine; FTY720, fingolimod; GelMA-G-MBGNs, mesoporous bioactive glass nanoparticles (MBGNs) with photo-cross-linkable GelMA which have been further integrated into GelMA; GelMA, gelatin derivative containing gelatin+ methacrylicanhydride; M, months; MBG-LGO, mesoporous bioactive glass- low graphene oxide; MBG- PHBHHx, (MBG)-doped poly(3-hydroxybutyrate-co-3-hydroxyhexanoate); MBG-HGO, mesoporous bioactive glass- high graphene oxide; MBG, mesoporous bioactive glasses; PHMB, BMP-2 + MBG-PHBHHx; PHMBD; BMP-2 + DMOG + MBG-PHBHHx; PHMD, DMOG + MBG-PHBHHx; PHMG, pure MBG-PHBHHx; VEGF, vascular endothelial growth factor; W, weeks. The bold values display the timing when each result was measured, and which method was used to do this*.

### Demographic Features of Animal Models

In total, 202 animals were surgically treated to form experimental models with critical-sized bone defects. Of these, 158 subjects were rats whilst Jia et al. (2015) used rabbit models. In 70 (34.7%) animals across three papers, a single defect (Jia et al., 2015; Qi et al., 2017; Jing et al., 2018) was surgically created, whilst the other 132 (65.3%) animals across six papers sustained two defects (Min et al., 2015; Zhao et al., 2015; Qi et al., 2017; Li et al., 2019; Wang et al., 2019; Wu et al., 2019). The length of time for which the scaffold was implanted before termination of the experiment ranged from 8 weeks to 3 months with one paper examining their findings at two end dates (both 4- and 8-weeks post-implantation) (Xin et al., 2017).

### Features of the Scaffolds

All studies included in this review used different bioactive glass compositions and/or glass fabrication methods to create them, as shown in Table 1. Eight (Min et al., 2015; Zhao et al., 2015; Qi et al., 2017; Xin et al., 2017; Li et al., 2019; Wu et al., 2019) out of the nine papers included added components to the bioactive glass scaffold. These studies can be divided into 3 broad categories. Firstly, those that incorporated additional elements into the bioactive glass network (Jia et al., 2015; Zhao et al., 2015; Wu et al., 2019). Secondly, those that used additives to the scaffold (Xin et al., 2017; Li et al., 2019; Wang et al., 2019). Finally, those that used biochemical factors (Min et al., 2015; Qi et al., 2017). These findings are summarized in Table 1.

### Do the Scaffolds Induce Osteogenesis?

In order to be included in this review, articles must have used micro-CT to evaluate osteogenesis. These results are detailed in Table 2. All nine papers showed evidence of bone regeneration when the bioactive glass was present, which, in the seven experiments where this was applicable (Jia et al., 2015; Min et al., 2015; Zhao et al., 2015; Xin et al., 2017; Jing et al., 2018; Li et al., 2019; Wu et al., 2019) was increased compared to the non-bioactive glass treated control.

Four papers assessed BMD as an indicator of osteogenesis. Both Zhao et al. (2015) and Wu et al. (2019) found that there was significantly increased bone formation seen on micro-CT in animals treated with bioactive glass scaffolds compared to negative controls or animals treated with scaffolds which did not include bioactive glass, respectively. Wang et al. (2019) and Qi et al. (2017) did not include a negative controls, with all experiments using bioactive glass. Both papers showed that BMD was seen to improve across all groups and was significantly increased in bioactive glass scaffolds treated with other substances, this has been further discussed below.

**Figure 2:**
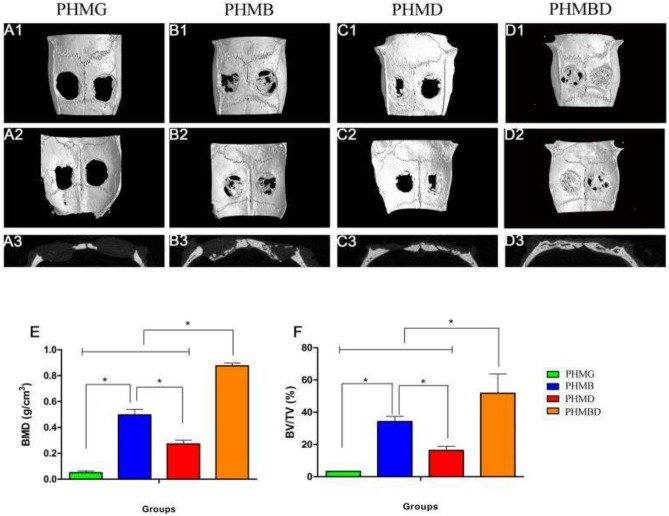
The effect of mesoporous bioactive glass containing groups on osteogenesis. The representative 3D reconstructions of superficial **(A1–D1)**, interior **(A2–D2)**, and sagittal images **(A3–D3)** of calvarial bone defects taken at 8 weeks after implantation are shown. Morphometric analysis of **(E)** BMD and **(F)** BV/TV is also shown as determined by micro-CT for each group at 8 weeks (*p < 0.05). MBG, mesoporous bioactive glasses; PHMB, BMP-2 + MBG-PHBHHx; PHMBD, BMP-2 + DMOG + MBG-PHBHHx; PHMD, DMOG + MBG-PHBHHx; PHMG, pure MBG-PHBHHx. Figure and caption reused from Qi et al. (2017). Used under the Creative Commons License (https://creativecommons.org/licenses/by/4.0/legalcode).

**Figure 3:**
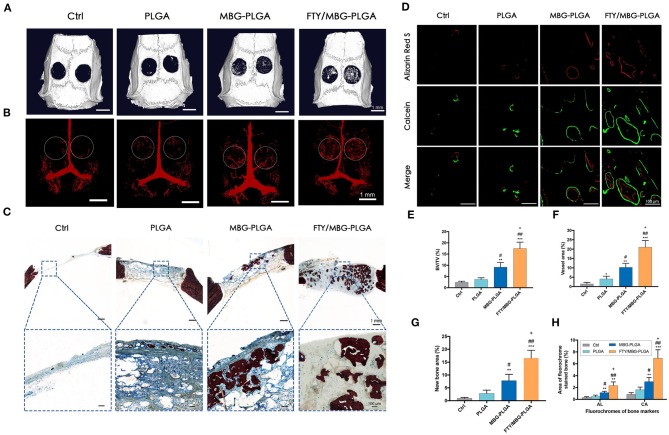
Osteogenesis and aniogenesis in critical sized calvarial bone defects. **(A)** Micro-CT images for the bone repair in calvarial defects of different groups after 8 weeks of implantation. **(B)** Micro-CT observation of the newly-formed blood vessels perfused with Microfil in the defect regions (as indicated by the white circle) at week 8. **(C)** New bone formation observed by Van Gieson's picrofuchsin staining at week 8 (red, new bone; blue, residual material). **(D)** Sequential fluorescent labeling observation for dynamic bone mineralization, Alizarin Red S (AL, week 4), and Calcein (CA, week 6). Quantitative data (*n* = 3) from micro-CT analysis of BV/TV, bone volume fractions **(E)** and blood vessel volume in the defect area **(F)**. The quantitative analysis of the new bone area of Van Gieson's picrofuchsin staining **(G)** and sequential fluorescent labeling **(H)**. *P < 0.05, **P < 0.01, ***P < 0.001 compared with control group; ^#^*P* < 0.05, ^##^*P* < 0.01 compared with PLGA group; ^+^*P* < 0.05 compared with MBG-PLGA group. *PLGA*, poly(lactic-co-glycolic acid); MBG-PLGA, mesoporous bioactive glass and poly(lactic-co-glycolic acid) scaffold; FTY/MBG-PLGA, FTY720 with mesoporous bioactive glass and=poly(lactic-co-glycolic acid) scaffold. Figure and caption reused from Li et al. (2019). Used under the Creative Commons License (https://creativecommons.org/licenses/by-nc-nd/4.0/legalcode).

Of the nine papers, only six used micro-CT to assess the bone volume fraction [bone volume/total volume (BV/TV)]. Wang et al. (2019) and Qi et al. (2017) found similar results as with BMD testing; again, the micro-CT assessment of bone volume fraction showed significantly increased osteogenesis in bioactive glass scaffolds treated with other substances. This is demonstrated in Figures 2E,F. The appearance of the calvarial bone defects at 8 weeks after implantation is shown in Figures 2A–D. Both Xin et al. (2017) and Wu et al. (2019) showed that the gels with bioactive glass nanoparticles showed significant increases in bone volume fraction compared to those treated without bioactive glass. Zhao et al. (2015) observed significantly greater bone volume fraction in MBG scaffolds group compared to the negative control group, with the Strontium- supplemented MBG scaffold showing significantly greater improvement than all other scaffolds. Li et al. (2019) found that FTY with bioactive glass and PLGA scaffolds showed the greatest increase in bone volume fraction compared to all other groups including the negative control. The MBG-PLGA group also showed a significantly greater increase bone volume fraction than the PLGA alone group as well as the negative control group. This effect on osteogenesis is demonstrated in Figures 3E,G. The micro-CT appearance of calvarial bone defects at 8 weeks is seen in Figure 3A whilst the blood vessels are seen in Figure 3B. The osteogenesis at week 8 is displayed by staining in Figure 3C. Figure 3D shows sequential fluorescent labeling to display the degree of bone mineralisation in each scaffold. Figure 3F shows quantitatively the degree of osteogenesis using staining and Figure 3H using fluorescent labeling.

Jia et al. (2015) found that, if left for 9 months, both the bioactive glass containing scaffolds, namely silicate 13–93 and borosilicate 2B6Sr, induced significantly more new bone formation compared to the negative control.

### Do the Scaffolds Induce Angiogenesis?

The studies included in this review assessed angiogenesis using two methods: immunohistochemistry to evaluate new blood vessel formation by staining vascular endothelium or by visualizing neo-vasculature using CT with Microfil® perfusion. These results are detailed in Table 2.

**Figure 4:**
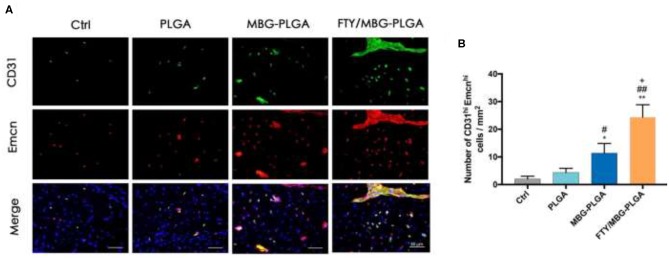
Angiogenesis in critical sized calvarial bone defects, using CD31^hi^ and Emcn^hi^ immunofluorescence staining for calvarial defect sections. **(A)** Immunofluorescence staining images of CD31 and Emcn for the calvarial defect sections from various groups (green, CD31; red, Emcn; blue, cell nuclei). **(B)** The quantitative analysis of CD31^hi^Emcn^hi^ cells per mm^2^ from the staining results, *n* = 5. *P < 0.05, **P < 0.01 compared with control group; ^#^*P* < 0.05, ^##^*P* < 0.01 compared with PLGA group; ^+^*P* < 0.05 compared with MBG-PLGA group. PLGA, poly(lactic-co-glycolic acid); MBG-PLGA, mesoporous bioactive glass and poly(lactic-co-glycolic acid) scaffold; FTY/MBG-PLGA, FTY720 with mesoporous bioactive glass and=poly(lactic-co-glycolic acid) scaffold. Figure and Caption reused from Li et al. (2019). Used under the Creative Commons License (https://creativecommons.org/licenses/by-nc-nd/4.0/legalcode).

Of the nine papers included in this review, seven used immunohistochemistry to investigate the angiogenesis-promoting qualities of bioactive glass (Jia et al., 2015; Qi et al., 2017; Xin et al., 2017; Jing et al., 2018; Li et al., 2019; Wang et al., 2019; Wu et al., 2019). The majority of the papers used CD31, where three out of five the included papers found that positively staining micro vessels were significantly increased in the experiments which included bioactive glass, in at least one time point (Xin et al., 2017; Jing et al., 2018; Li et al., 2019). This effect, in the study by Li et al. (2019), is demonstrated in Figures 4A,B. Notably, Jia et al. (2015) found that although there was a significant increase in density between the 2B6Sr containing scaffold compared to the 13-93 and ABG groups at 3 months, this difference was not observed at 9 months. Similarly, Qi et al. (2017) observed that although neovascularization and CD31 staining was seen in all bioactive glass treated groups, this was increased in BMP-2+DMOG+MBG-PHBHHx scaffolds and PHMD compared to the PHMB and PHMG, thus suggesting that DMOG helps to increase angiogenesis. Three other stains were also used, each by a single paper. Similar to their CD31 findings, Jing et al. (2018) observed that the number of VEGF-positive blood vessels were significantly higher in bioactive glass treated experiments compared to negative controls, suggesting higher levels of angiogenesis with bioactive glass. In contrast, Wang et al. (2019) found limited CD34 staining in bone defects treated with bioactive glass alone, whilst there was more staining as the concentration of graphene oxide used increased. However, there was no negative control used in this study for comparison. Similarly, Wu et al. (2019) found similar smooth muscle (α-SMA) antigen staining in the bioactive glass containing group (BG/CH/SF/GP) compared to the non-bioactive glass containing group (CH/SF/GP).

In summary, the addition of bioactive glass alone increased angiogenesis marker staining in four of nine papers (Jia et al., 2015; Xin et al., 2017; Jing et al., 2018; Li et al., 2019), but this was not significant in the experiments by Wu et al. (2019) and can't be assessed with Wang et al. (2019) as they did not test the effect of bioactive glass alone. Five out of nine papers included in this review assessed angiogenesis through analyzing Microfil® perfusion studies (Min et al., 2015; Zhao et al., 2015; Qi et al., 2017; Li et al., 2019; Wang et al., 2019). All of these papers showed evidence of angiogenesis and the formation of new blood vessels in the bone defect, although this process could be enhanced by the addition of other substances. Notably, both Li et al. (2019) and Zhao et al. (2015) showed that scaffolds containing bioactive glass induced significantly greater new blood vessel area than those without.

### Studies Reporting Relative Increase in Angiogenesis and Osteogenesis

Five out of nine studies (Min et al., 2015; Zhao et al., 2015; Xin et al., 2017; Jing et al., 2018; Li et al., 2019) showed increases in both angiogenesis and osteogenesis in bioactive glass-containing scaffolds compared to scaffolds without bioactive glass (Xin et al., 2017; Li et al., 2019) or negative control groups (Min et al., 2015; Zhao et al., 2015; Jing et al., 2018).

Wu et al. (2019) showed that, although the bioactive glass containing scaffolds showed increased levels of osteogenesis compared to those without bioactive glass, they did not always show an increase in angiogenesis compared to those without. Jia et al. (2015) showed that although both bioactive glass containing scaffolds showed complete more osteogenesis than in the negative control, there was no comment on the extent of angiogenesis in the negative control for comparison. As all groups contained bioactive glass, the results of Qi et al. (2017) and Wang et al. (2019) cannot be commented on in this respect.

### Effects of Changing the Composition of the Bioactive Glass Scaffolds

Seven out of the nine papers included in this review studied bioactive glass scaffolds with added non-cellular components as outlined below. These can be broadly divided into components which were incorporated into the bioactive glass network, additives, and finally biochemical factors.

#### Incorporation of Additional Elements Into Bioactive Glass Network

Ions and inorganic compounds: Three papers investigated the effect of incorporating metals into the bioactive glass network. Jia et al. (2015) added boron trioxide (borosilicate) to the bioactive which made for very successful scaffolds. Increased osteogenesis compared to the negative control was observed for both silicate and borosilicate scaffolds, whilst for angiogenesis, the borosilicate-containing bioactive glass scaffold showed higher levels of CD31 positively stained vasculature initially, but by 9 months there was no difference between the two scaffolds. Zhao et al. (2015) also found that although the addition of mesoporous glass showed more favorable outcomes with both osteogenesis and angiogenesis, however, the addition of strontium improved outcomes in terms of bone growth, bone volume fraction and angiogenesis. Similarly, Wu et al. (2019) found that the addition of copper allowed for a full repair of the bone defect, whilst bioactive glass alone only allowed for partial repair. Furthermore, α smooth muscle (α-SMA) antigen staining was greater in copper treated scaffolds. Micro-CT results further corroborated these results with copper improving osteogenesis as observed on micro-CT, significantly higher BMD and BV/TC.

#### Use of Additives to the Bioactive Glass Scaffold

Wang et al. (2019) examined the effects of adding graphene oxide to bioactive glass scaffolds. Micro-CT showed markedly increased osteogenesis in groups treated with higher concentrations of graphene oxide in the bioactive glass. Moreover, angiogenesis was also seen to be increased as shown by CD34 staining and Microfil® perfusion experiments in bioactive glass containing graphene oxide compared to those without.

Two papers evaluated the effect of bioactive glass when used in combination with organic polymers to form a scaffold. Xin et al. (2017) found that crossing linking MBGNs with photo-cross-linkable GelMA and then incorporating GelMA increased angiogenesis, as shown by significantly higher levels of CD31 staining at 4 weeks compared to scaffolds just created by chemically modifying mesoporous bioactive glass nanoparticles with photo-cross-linkable GelMA. Furthermore, Li et al. (2019) found that MBG-PLGA composite scaffolds were more effective at inducing osteogenesis when the bioactive lipid, FTY720, was added, as shown by micro-CT study. Microfil® perfusion studies and CD31 staining showed a similar effect on angiogenesis.

#### Use of Biochemical Factors

Two out of the nine papers included in this paper assessed the effect of the addition of osteoinductive biochemical factors. One of the factors was DMOG. Qi et al. (2017) found that BMD was highest in the bioactive glass group treated with DMOG as well as BMP-2 along with angiogenesis as observed with CD31 staining. In experiments by Min et al. (2015) angiogenesis was observed with bioactive glass with or without the addition of DMOG. However, DMOG loaded scaffolds showed an advantage as they promoted the ingrowth of dense vessels into the center of the defect, whilst unloaded bioactive glass scaffolds encouraged growth around the periphery.

## Discussion

The regenerative capacity of bioactive glass scaffolds are dependent on a number of factors including the composition of the bioactive glass scaffolds, the method of fabrication and the microstructure of the scaffold amongst others (El-Rashidy et al., 2017). Although bioactive glass could enhance bone formation, more research is needed to characterize the influencing factors. This review attempts to elucidate the effects of non-cellular bioactive glass-containing scaffolds on osteogenesis and angiogenesis in bone defect by analyzing the *in vivo* animal literature.

### Osteogenesis and Bioactive Glass Scaffolds

Osteogenesis was initially thought to take place through the dissolution of particles (in the original 45S5 Bioglass® causing an HCA layer to form rapidly, the glass degrading and then allowing space for bone ingrowth Hench and Jones, 2015. It was later shown that the dissolution of the bioactive glass particles releases ions that acted as signals into the cells, prompting upregulation of certain genes and subsequent increases in nuclear transcription factors, cell cycle regulators, and growth factors such as insulin-like growth factor II (IGF-II) (Xynos et al., 2000, 2001). Transcription of extracellular matrix (ECM) components and secretion into a mineralized matrix is thought to lead to the formation and growth of bone nodules along with differentiation of the osteocyte phenotype (Hench and Jones, 2015). Individual ions also play a role, with ions such as calcium being important for upregulation of osteogenic genes (Hench, 2009) and silicon important for the formation and calcification of bone tissue (Carlisle, 1981).

#### Bone Volume Fraction as an Indicator for Osteogenesis

Two studies (Qi et al., 2017; Wang et al., 2019) found that bone volume fraction significantly increased in bioactive glass scaffolds treated with other substances. Xin et al. (2017) and Wu et al. (2019) showed that gels with bioactive glass nanoparticles also had significant increases in bone volume fraction compared to defects treated without bioactive glass. Similarly, Li et al. (2019) showed that the bioactive glass containing groups showed greater increases in the bone volume fraction than those without bioactive glass. Zhao et al. (2015) found a greater bone volume fraction in MBG scaffolds than negative controls. Jia et al. (2015) found that bioactive glass containing scaffolds induced more bone formation than negative controls.

#### BMD as an Indicator for Osteogenesis

Wang et al. (2019) and Qi et al. (2017) found that bioactive glass scaffolds increased BMD. Zhao et al. (2015) and Wu et al. (2019) found that BMD also increased in animals with bioactive glass scaffolds compared to negative controls or animals with scaffolds without bioactive glass. Thus, these results on the whole do indicate that bioactive glass scaffolds could induce osteogenesis, with no papers showing an insignificant difference between control and bioactive glass containing groups.

### Angiogenesis and Bioactive Glass Scaffolds

Angiogenesis is the growth of blood vessels from existing vasculature, occurring throughout life and can be physiological or pathological. The two types of angiogenesis are sprouting and intussusceptive. Intussusceptive angiogenesis is when a vessel is split into two and is more relevant in embryogenesis than in the growth of vascular networks into scaffolds. Sprouting angiogenesis is initiated in hypoxic conditions due to the expression of VEGF-A by parenchymal cells and a tip cell guided by filopodia will follow the VEGF gradient using VEGF receptors. Vacuoles will form and tip cells will fuse forming a continuous lumen for blood to flow through and once oxygen is supplied, VEGF-A levels return to normal. Other signaling pathways such as the delta-notch pathway are also responsible for sprout formation, but even this pathway is reliant on VEGF expression demonstrating the importance of the factor (Adair and Montani, 2010). Markers such as CD31 which is an endothelial cell-cell adhesion molecule have been shown to be an indication of new vessel formation (DeLisser et al., 1997), as well as CD34 which is expressed on tip cells (Siemerink et al., 2012) and a-SMA which is an indication of microvessel density (Tonino and Abreu, 2011).

One of the indications for the development of bioactive glass scaffolds, was the potential for enhanced angiogenesis. The creation of a vascular network is vital for tissue to successfully regenerate and has been acknowledged as a significant issue in tissue engineering, particularly for larger tissues such as bone. Spontaneous growth of vessels has a slow rate and vascularization of an implant may take weeks, during which time tissue is unevenly supplied and may centrally go hypoxic (Rouwkema et al., 2008). Bioactive glass has been shown to be proangiogenic *in vitro*, stimulating the expression of VEGF from fibroblasts and proliferation of microvascular endothelial cells (Day, 2005). The ionic dissolution products such as Si, which were shown to affect gene expression in human osteoblasts explaining osteogenic effects (Xynos et al., 2001), have also been shown to be pro-angiogenic (Zhai et al., 2012). Calcium silicate has been shown to stimulate VEGF and VE-cad expression, with silicon ions determined to have a significant role in angiogenesis by dilution experiments and calcium ions supplementing the silicon's effect (Li and Chang, 2013).

The results from papers on the effects on bioactive glass on angiogenesis *in vivo* are mixed. Four papers showed evidence of bioactive glass alone increasing immunohistochemistry staining indicating angiogenesis e.g., CD31 (Qi et al., 2017; Xin et al., 2017; Jing et al., 2018; Li et al., 2019), VEGF (Jing et al., 2018). Increased Microfil® perfusion was seen by the only four groups who tested Microfil® perfusion for controls or bioactive glass alone as part of their study (Min et al., 2015; Zhao et al., 2015; Qi et al., 2017; Li et al., 2019). Wang et al. (2019) did not compare to control but showed limited staining of CD34 with bioactive glass alone, whilst Wu et al. (2019) saw no difference in a-SMA staining between non-bioactive glass and bioactive glass treated groups. Jia et al. (2015) found no significant difference in CD31 staining between bioactive glass scaffolds (13–93 silicate alone) and autologous bone graft controls at 3M and 9M. This is significant as the three other studies that used CD31 as a marker and compared angiogenesis to a non-bioactive glass control all found an increase (Xin et al., 2017; Jing et al., 2018; Li et al., 2019), but Jia et al. (2015) was also the only paper that used a rabbit femoral defect instead of rat skull. Although the papers tend to indicate that bioactive glass has pro-angiogenetic properties, the results are not as overwhelming as the osteogenic effects and indicate a potential avenue for future improvement, which will be discussed below.

### How Does Changing the Composition of Bioactive Glass Scaffolds Impact Their Properties?

Only a single study reviewed focused on the use of bioactive glass without any non-cellular additives (Jing et al., 2018). Innovative scaffolds tend to involve the incorporation of metals, biochemical factors and organic compounds. It is important to acknowledge this variance in composition as the heterogeneity of the scaffolds makes it difficult to directly compare results.

#### Incorporating Additional Elements (Boron, Strontium, Copper) Into the Bioactive Glass Network

Boron seems to have a physiological role in bone where it accumulates, with recent mechanisms proposed such as simultaneous activation of the NaBC1 ion channel and VEGFR (Moseman, 1994). There is also evidence in other models that boron in bioactive glass allows for enhanced bone formation, potentially due to its impact on angiogenesis (Gorustovich et al., 2006). Jia et al. (2015) found boron improved angiogenesis (assessed by CD31 staining) at 3 months compared to standard silicate bioactive glass, but this difference was not found at 9 months. This suggests that boron can increase angiogenesis early but there is no overall effect by the end of 9 months. Zhao et al. (2015) found strontium both increased BMD and bone volume fraction on micro-CT as well as Microfil® perfusion, therefore having osteogenic and angiogenic benefits. This follows on from evidence that strontium stimulates bone formation and reduces bone resorption (Kyllönen et al., 2015). This could be due to strontium ions' ability to inhibit receptor activator of nuclear factor kappa-B ligand (RANKL) in mesenchymal stem cells, thereby inhibiting osteoclast differentiation (Marie et al., 2011). Strontium also promotes vessel formation by stimulation of VEGF, MMP-2 etc. (Zarins et al., 2017). Copper also increased osteogenic and angiogenic effects of bioactive glass, particularly angiogenesis (a-SMA) which was similar between controls and standard bioactive glass (Wu et al., 2019). A link between copper and bone health has been established previously (Qu et al., 2018) and has been found to have osteostimulation properties, facilitating new bone formation (Wu et al., 2013b). The ion also stimulates angiogenic factor expression such as VEGF and HIF-1 (Du et al., 2018). Copper and silicon ions have also been reported to have synergistic effects on angiogenesis *in vitro* (Kong et al., 2014).

#### Using Additives (Graphene Oxide and Organic Polymers) in Bioactive Glass Scaffolds

Wang et al. (2019) used graphene oxide, an inorganic compound, and found that not only did the addition increase osteogenesis and angiogenesis (assessed by both CD34 and Microfil® perfusion), but a high amount had significantly better results than a low amount of graphene in the scaffold. This was particularly evident in the CD34 staining, which was limited in the use of standard bioactive glass scaffolds. Graphene oxide is a conductive material, which are known to enhance bone repair, and it is known to induce osteoblastic differentiation and mineralization (Cheng et al., 2018).

MBG-PLGA (PLGA alone was equivalent to control) composites were evaluated by Li et al. both with and without FTY720, a bioactive lipid. FTY720 is used as an immunosuppressive drug for multiple sclerosis due to a reduction in peripheral lymphocytes and T cell inhibition (Baer et al., 2018) with a developing role in cancer therapy (White et al., 2016). There is evidence local delivery of FTY720 in calvarial defects increases bone volume (Huang et al., 2012), potentially by stimulating sphingosine 1-phosphate receptors and as a result, the local microvascular network (Aronin et al., 2010). Those with FTY had increased bone volume, CD31 staining and Microfil® blood vessel area implying a benefit of this organic compound. Xin et al. (2017) incorporated photo-cross-linked GelMA into a bioactive glass nanoparticles scaffold in which GelMA was part of the composition of the particles. GelMA has good biocompatibility and is similar to the ECM, as a semi-synthetic hydrogel. There was no control without any GelMA at all, but the addition of photo-cross-linked GelMA to the composite nanoparticles did increase the bone volume fraction and CD31 staining. It is important at this point to acknowledge the use of nanoparticles in bioactive glass. Their high surface to volume ratio and ability to incorporate into matrices more evenly helps maximize the yield of the bioactive glass in bone regeneration (Tabia et al., 2019).

#### The Use of Biochemical Factors (DMOG) in Bioactive Glass Scaffolds

DMOG is an osteoinductive biochemical factor. It is a competitive inhibitor of prolyl hydroxylase enzymes which subsequently leads to less degradation of the angiogenic factor HIF-1a (Zhang et al., 2016). The osteogenic effects of DMOG are not clear as Qi et al. (2017) found BMD was better than bioactive glass alone but for Min et al. (2015) there was no difference. Nevertheless, both papers found a benefit in angiogenesis. Qi et al. (2017) saw CD31 staining and Microfil® perfusion was greatest with both DMOG and bioactive glass, but interestingly DMOG alone had a significantly better effect than bioactive glass alone, which is the opposite to osteogenic results. Min et al. (2015) also found that DMOG-loaded bioactive glass scaffolds showed more vessel ingrowth into the center of the defect compared to bioactive glass alone as opposed to around the peripheries.

### Other Bioactive Glass Scaffolds and the Future

For this systematic review, any scaffolds with cells added were excluded due to the potentially drastic impact on bone formation. Nevertheless, scaffolds now frequently incorporate stem cells to enhance the potential for regeneration. Adipose-derived mesenchymal stem cells are commonly the cells used (Handel et al., 2013; Du et al., 2018) due to the relative ease in obtaining them and potential for differentiation into multiple relevant cell types (Strem et al., 2005). Other stem cell types used include those derived from bone and umbilical cords, with benefits in particular seen with bone-marrow derived stem cells for osteogenesis and umbilical cord for angiogenesis on comparison (Kargozar et al., 2018b). UC-derived stem cells may therefore be useful in stimulating angiogenesis when added onto bioactive glass, which as seen from above has a bigger need to be targeted than osteogenesis.

The future of bioactive glass in bone regeneration is vast. The biocompatibility and osteogenic effects make it ideal to encourage the healing of bone defects, as seen both *in vitro* and reviewed here *in vivo*. One avenue that is being explored in bioactive glass currently is the controlled release of biomolecules from the scaffold. This could be utilized in bone regeneration with targeted drug delivery. The factors released could include transforming growth factors, bone morphogenetic proteins, stromal cell-derived factors etc. to imitate *in vivo* signaling for repair, many of which are approved for use in humans and incorporated into implants already (Gothard et al., 2014; Baino et al., 2018). The other key advancement in bioactive glass scaffolds, is the structure of these scaffolds. Mesoporous bioactive glass scaffolds were initially brittle meaning they could not be used in practice. Developments in 3D printing and polymer addition have strengthened the scaffold (Baino et al., 2018), but structure could still be improved, with recent advancements in nanoparticles increasing the surface area exposed to tissue. A randomized controlled clinical trial in humans using bioglass for bone healing after tooth extraction showed preservation of alveolar bone and enhanced bone remodeling (El Shazley et al., 2016). Despite this being a very different situation to critical size bone defects, the successful use of the biomaterial in humans implies promise for bioactive glass in therapeutics.

### Limitations

It is important to highlight the limitations of this systematic review. First, only three databases were searched (Pubmed, Scopus, and EMBASE). Additionally, only studies that were in English were looked at, therefore there might have been some relevant studies that may have been overlooked. Nine studies were included in this review, which represents a very small number in the bioactive glass scaffold literature. The studies that were included used a variable type of scaffold and used different animal models limiting any direct comparison between studies. There was also variability in the methodology of the studies, for example some studies using BMD as opposed to bone volume fraction as a marker for osteogenesis, and different immunohistochemistry markers for angiogenesis. Future studies using standardized methods of measuring these properties would help in comparison and making conclusions. Further, some studies did not include a control, making any definitive causal conclusions hard to ascertain, as well as only one study evaluating results at a longer time point of 9 months (Jia et al., 2015). Future studies should aim to take measurements at more than one time point and indeed in the long term, which could be important for clinical translation of such scaffolds from animal defect models to humans.

## Conclusion

The data from this systematic review suggests that despite the heterogeneity of the scaffolds and markers assessed, there is consistent evidence that bioactive glass does improve bone regeneration in these models, both by osteogenic and angiogenic measures. This review also highlighted the benefits of changing the composition of the bioactive glass scaffolds using 3 methods. Firstly, incorporation of additional elements into the bioactive glass network such as boron, copper and strontium. Secondly, the use of additives and finally the use of biochemical factors. However, comparisons between the scaffolds are limited by the heterogeneity of study methods. The future of bioactive glass will broaden as advancements in technology are made and the structure, composition and additives for bone scaffolds must be optimized for and trialed in humans, which will take a significant amount of research and time. Increased consistency in composition of scaffold and outcome measures will help in determining the scaffold with optimal results. Nevertheless, the *in vivo* results do confirm that bioactive glass has a future in bone regeneration.

## Author Contributions

CDSR, CKIR, JR, DS, and WK: conceptualization, methodology, and writing—review and editing. CDSR, CKIR, JR, and DS: writing—original draft preparation. WK: supervision.

### Conflict of Interest

The authors declare that the research was conducted in the absence of any commercial or financial relationships that could be construed as a potential conflict of interest.

## Abbreviations

45S5: glass with 45 wt.% of SiO_2_ and 5:1 molar ratio of calcium to phosphorous
α-SMA: alpha-smooth muscle actin
BMD: bone mineral density
BMP-2: human bone morphogenetic protein-2
BV/TV,: bone volume/total bone volume i.e. bone volume fraction
CD31: cluster of differentiation 31
CD34: cluster of differentiation 34
Cu-BG/CH/SF/GP: copper-doped bioactive glass with chitosan/silk fibroin/glycerophosphate concentration 1
Cu-BG/CH/SF/GP(II): copper-doped bioactive glass with chitosan/silk fibroin/glycerophosphate concentration 2
DMOG: dimethyloxallyl glycine
ECM: extracellular matrix
FTY720: fingolimod
GelMA: gelatin methacryloyl
GelMa-G-MBGNs: MGBN with photo-cross-linkable GelMA which have been further integrated into GelMA
HCA: hydroxycarbonated apatite
HIF-1: hypoxia-inducible factor-1
HIF-PH: hypoxia-inducible factor prolyl hydroxylase
IGF-II: insulin-like growth factor II
M: months
MBG: mesoporous bioactive glass
MBGN: mesoporous bioactive glass nanoparticle
MBG-LGO: MBG-low graphene oxide
MBG-HGO: MBG-high graphene oxide
MBG-PHBHHx: MBG-doped poly(3-hydroxybutyrate-co-3-hydroxyhexanoate)
Mm: millimeters
MMP-2: matrix metallopeptidase-2
MPHS: MBG with poly(3-hydroxybutyrate-co-3-hydroxyhexanoate)
PHMB: BMP-2 + MBG-PHBHHx
PHMBD: BMP-2 + DMOG + MBG-PHBHHx
PHMD: DMOG + MBG-PHBHHx
PHMG: pure MBG-PHBHHx
PLGA: poly(lactic-co-glycolic acid)
RANKL: receptor activator of nuclear factor kappa-B ligand
rhBMP-2: recombinant human bone morphogenetic protein-2
TEOS: tetraethyl orthosilicate
TEP: triethyl phosphate
VEGF: vascular endothelial growth factor
W: weeks
Wt: weight.
